# Correction: The bZIP transcription factor AREB3 mediates FT signalling and floral transition at the Arabidopsis shoot apical meristem

**DOI:** 10.1371/journal.pgen.1010920

**Published:** 2023-08-29

**Authors:** Damiano Martignago, Vítor da Silveira Falavigna, Alessandra Lombardi, He Gao, Paolo Korwin Krukowski, Massimo Galbiati, Chiara Tonelli, George Coupland, Lucio Conti

The fifth author’s name is misspelled. The correct name is Paolo Korwin Krukowski. The correct citation is:

Martignago D, da Silveira Falavigna V, Lombardi A, Gao H, Korwin Krukowski P, Galbiati M, et al. (2023) The bZIP transcription factor AREB3 mediates FT signalling and floral transition at the Arabidopsis shoot apical meristem. PLoS Genet 19(5): e1010766. https://doi.org/10.1371/journal.pgen.1010766
