# Supplementary figures and images for: The bZIP transcription factor AREB3 mediates FT signalling and floral transition at the Arabidopsis shoot apical meristem
Source: PLoS Genet. 2023 May 15;19(5):e1010766. doi: 10.1371/journal.pgen.1010766 (PMC10212096; doi:10.1371/journal.pgen.1010766)

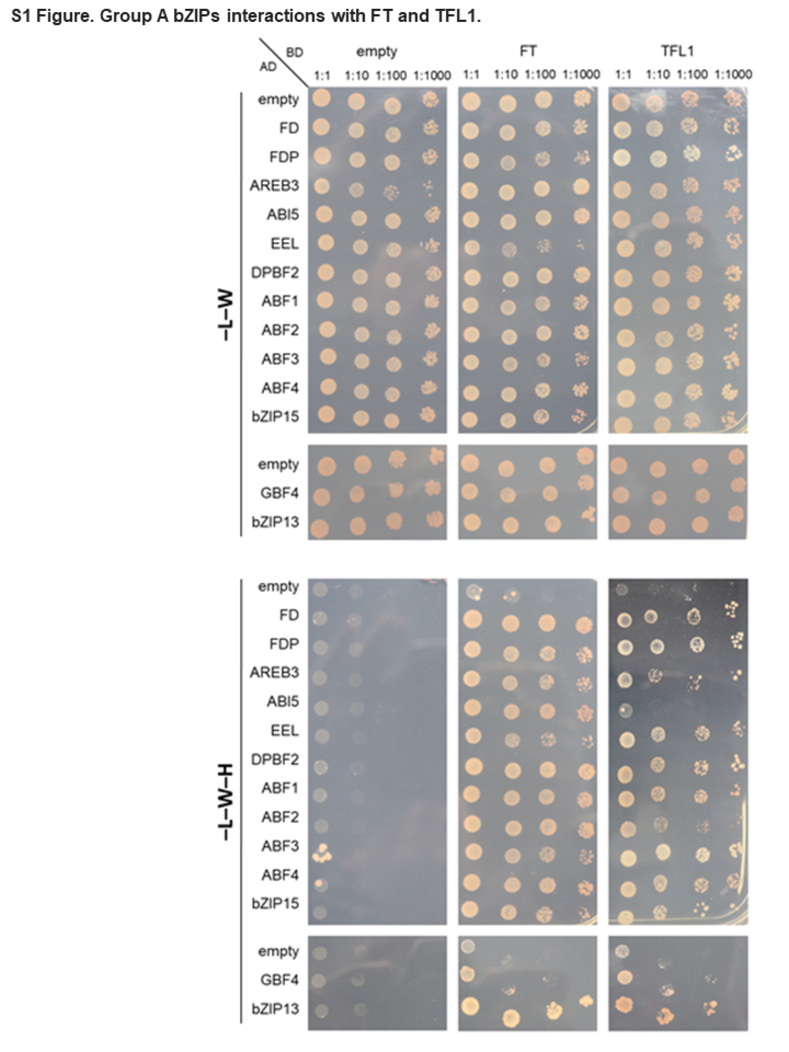

Supplement: S1 Fig — Y2H assays testing protein interactions among group A bZIP TFs and the PEBP proteins FT and TFL1. The panel present in Fig 1 was extracted from the 1:1 dilution column. (TIF) [file pgen.1010766.s004.tif]

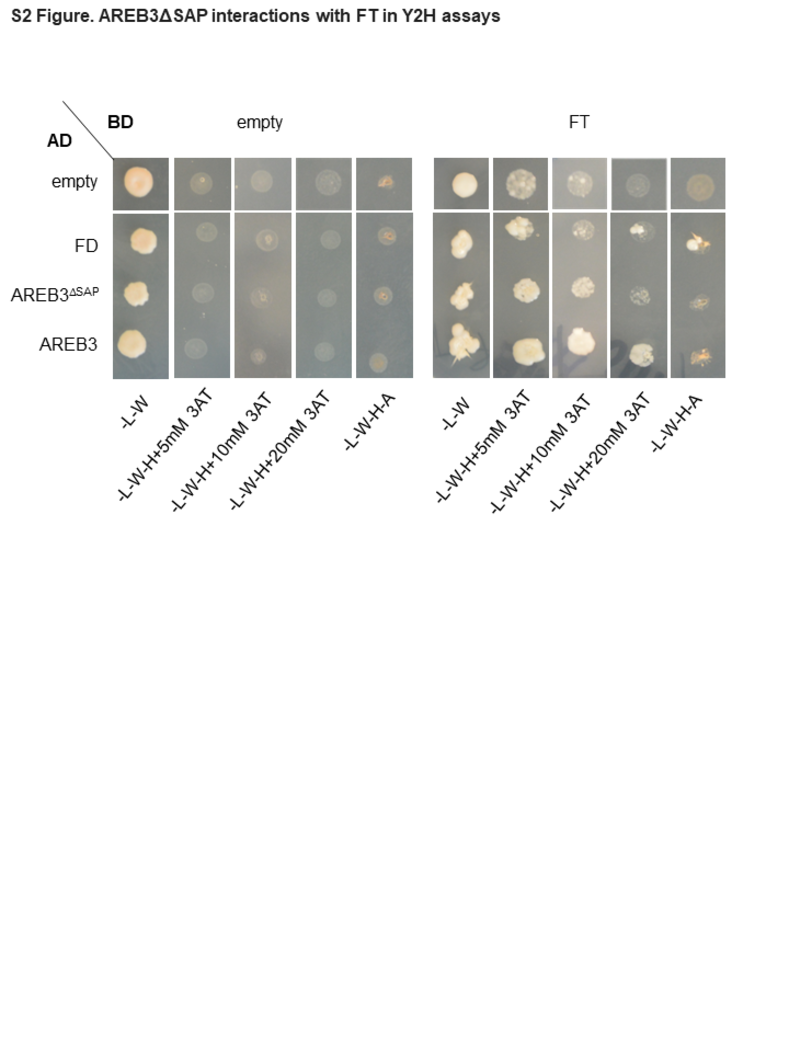

Supplement: S2 Fig — Y2H assays testing protein interactions between FT and the bZIP TFs FD and AREB3. In the AREB3ΔSAP construct, the SAP motif was completely removed and a stop codon was inserted after R290 (R291*). AREB3 interaction with FT is weakened but not suppressed by the lack of the SAP motif. (TIF) [file pgen.1010766.s005.tif]

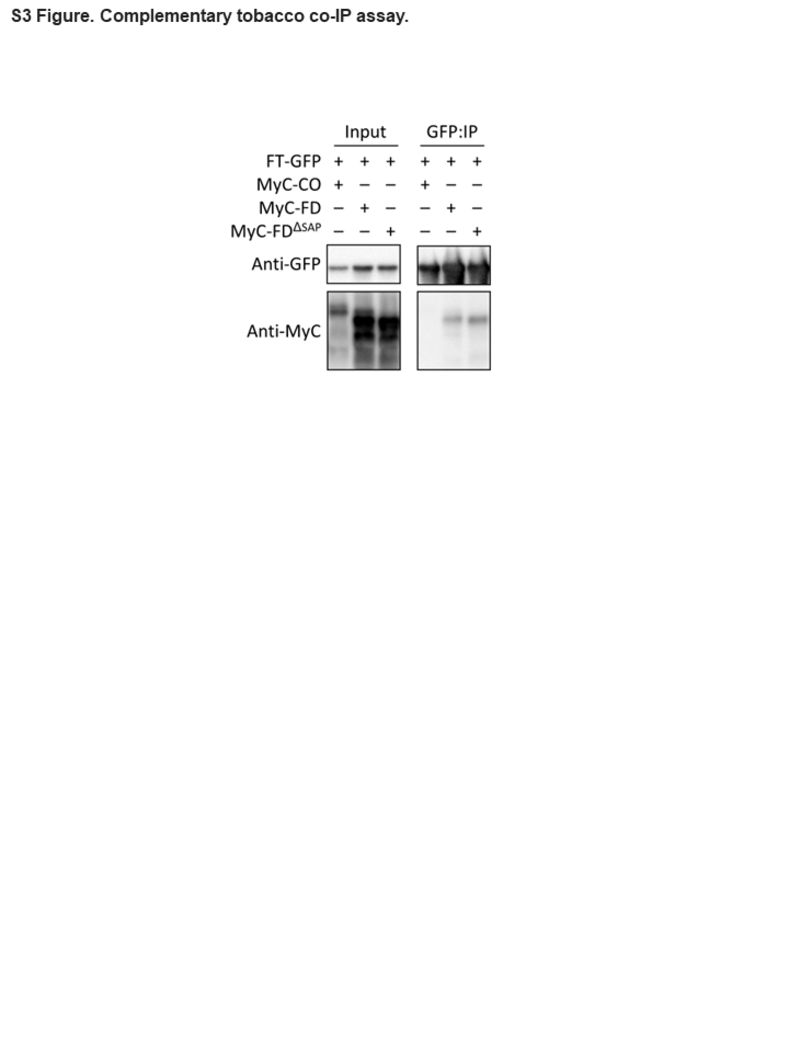

Supplement: S3 Fig — N. benthamiana co-IP of protein interactions among wt FD and FDΔSAP with FT. CO was used as a negative control for the interaction with FT. Pairwise protein–protein interactions were tested by co-agroinfiltration of tobacco leaves. CO and both FD versions were translationally fused to MyC, whereas FT was translationally fused to GFP. The input was composed of total proteins recovered before the IP. GFP-fused proteins were pulled down using anti-GFP nanobody (VHH) beads and immunoblotted using anti-MyC or anti-GFP antibody. (TIF) [file pgen.1010766.s006.tif]

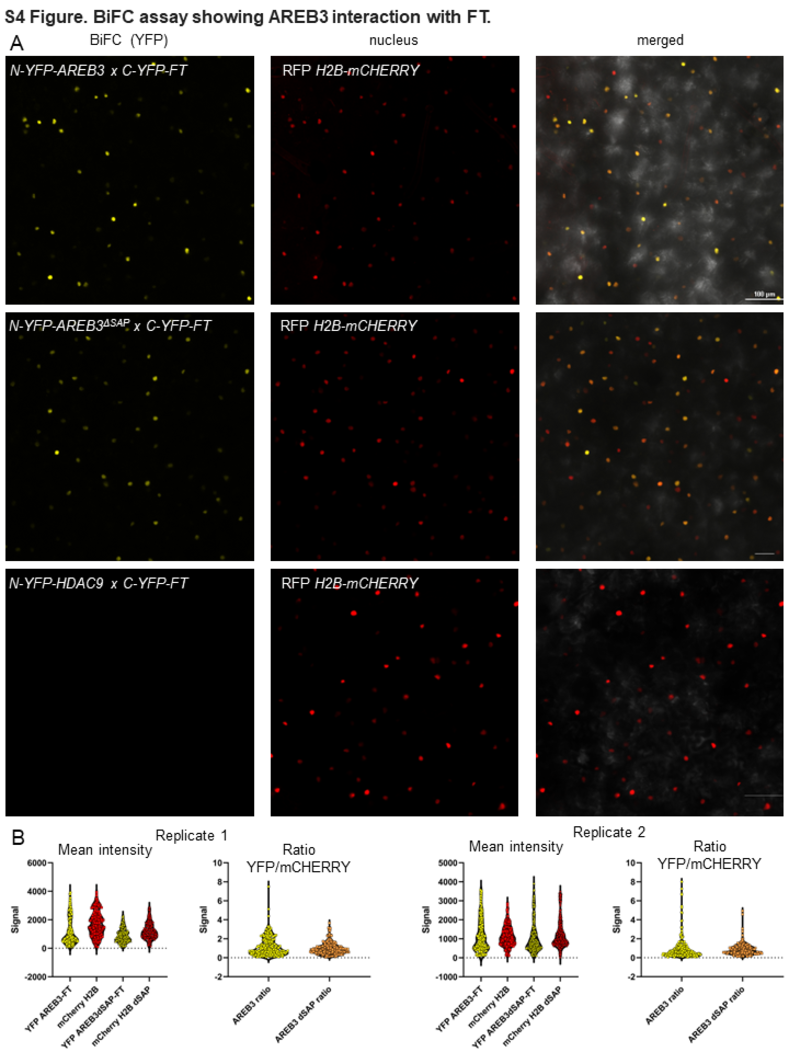

Supplement: S4 Fig — (A) BiFC assays testing protein interactions between AREB3 or AREB3ΔSAP and FT. N. benthamiana plants were co-infiltrated with vectors expressing mCHERRY-tagged nuclear protein H2B as a nuclear marker, C-YFP-FT, and either one of N-YFP-AREB3, N-YFP-AREB3ΔSAP or the nuclear protein HDA19 as negative control. Z-stack maximum projections are shown in the pictures. Scale bar 100μm. (B) Semiquantitative analysis of BiFC interactions. The average fluorescence of the nuclei expressing interacting proteins was quantified on n>150 nuclei in two independent replicates. Absolute YFP and mCHERRY signals were measured and the YFP/mCHERRY ratio was calculated. No significant differences were observed between AREB3 and its truncated version AREB3ΔSAP (R291*) lacking the SAP motif. (TIF) [file pgen.1010766.s007.tif]

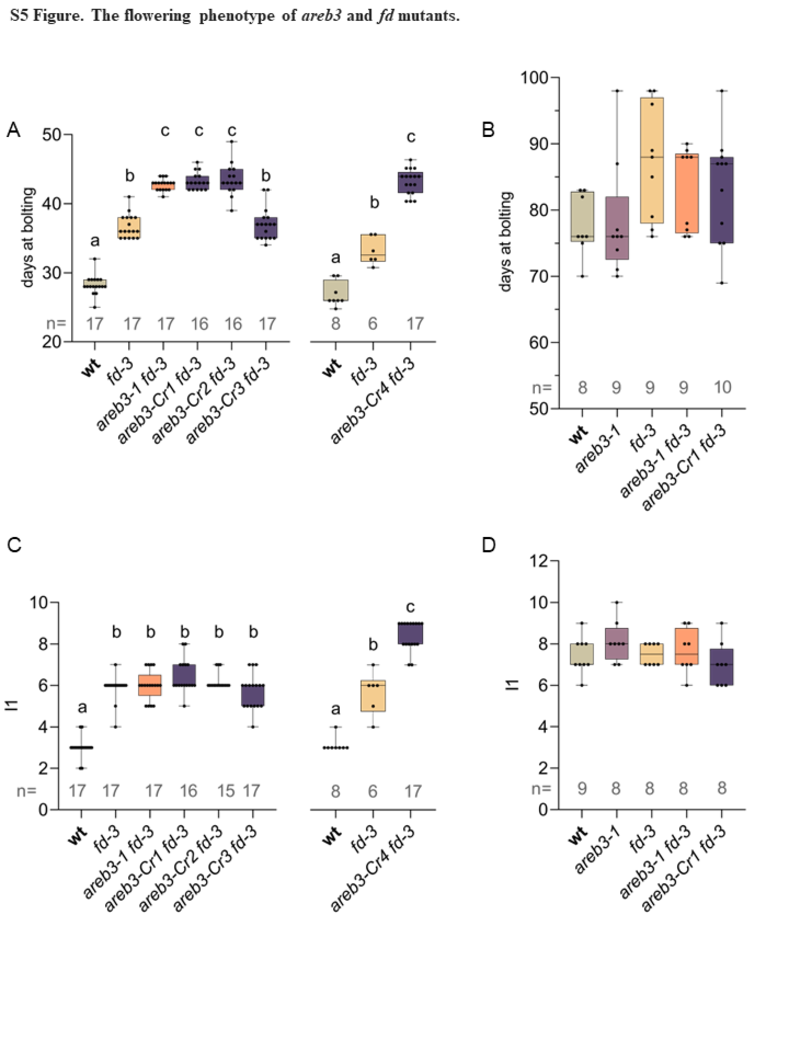

Supplement: S5 Fig — (A) Days at bolting of the indicated genotypes grown under LDs, measured as number of days between sowing date and the date in which the plants presented a >5mm high floral bolt. Significance a vs b, c p = 4.89e-10; b vs c p = .89e-10 (left panel); a vs b, c p<2.63e-6; b vs c p = 1.67e-11 (right panel). (B) Days at bolting of plants grown in SD, data analysis revealed no significant differences. (C) Number of cauline leaves (I1 phase) of plants grown under LDs. Significance a vs b p = 4.89e-10 (left panel); d vs e, f p<4.17e-7; e vs f p = 1.36e-8 (right panel). (D) I1 of plants grown under SDs, revealed no significant differences. (TIF) [file pgen.1010766.s008.tif]

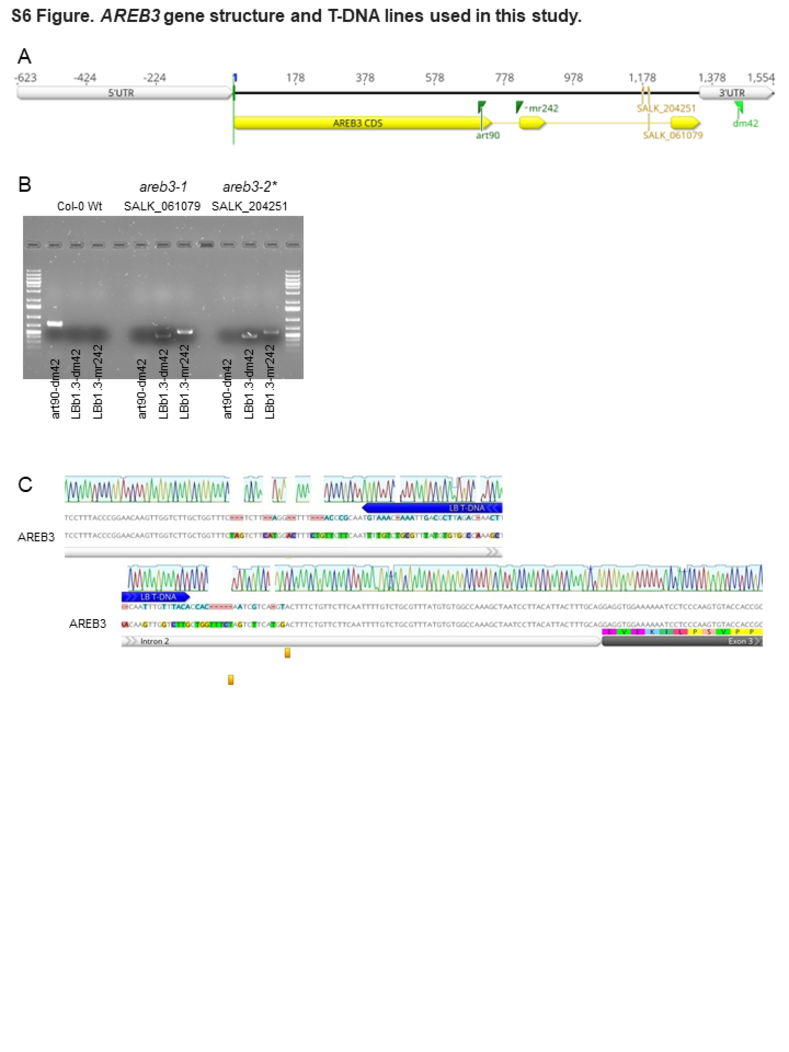

Supplement: S6 Fig — (A) Schematic representation of the AREB3 gene, the position of T-DNA insertions (and corresponding SALK identifier), and the position of the primers used (green). (B) Agarose gel showing amplification of genomic DNA extracted from wt, SALK_061079 and SALK_204251 homozygous plants. Note that both SALK lines show the same amplification pattern using primers on T-DNA left border (LBb1.3) and primers both upstream (mr242) and downstream (dm42) of the putative insertion side. This shows that SALK_061079 and SALK_204251 lines contain the same T-DNA insertions. (C) Sequencing of the amplicons using primers mr242 and dm42, respectively, allowed the fine mapping of the T-DNA insertion site. The T-DNA is inserted between Chr3:21046624 and Chr3:21046636 in the intron 2 of the AREB3 cds and does not affect any exon. Images produced using Geneious version 2022.0 created by Biomatters. (TIF) [file pgen.1010766.s009.tif]

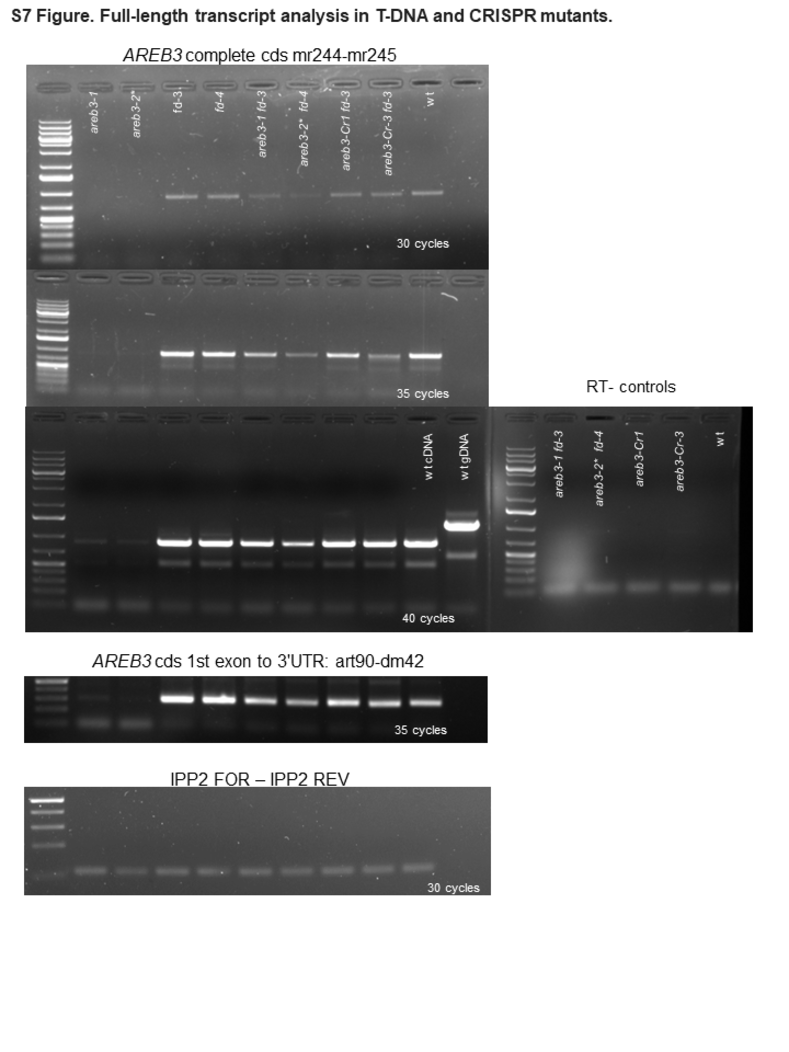

Supplement: S7 Fig — RT-PCR on cDNA from a pool of 5 whole seedlings grown for 2 weeks in 1/2MS agar plates under LD conditions. The primers used (mr244-mr245) amplify the whole cds. Residual accumulation of full length AREB3 transcript is visible in the double mutants of areb3-1 fd-3 and areb3-2* fd-4 starting from 30 cycles of amplification. At 40 cycles, a residual expression is visible as a faint band also in single areb3-1 and areb3-2* mutants. Similar results were obtained using a primer pair that amplifies a fragment starting at the 3’-terminal portion of the 1st exon and ending 124bp into the 3’UTR (art90-dm42). (TIF) [file pgen.1010766.s010.tif]

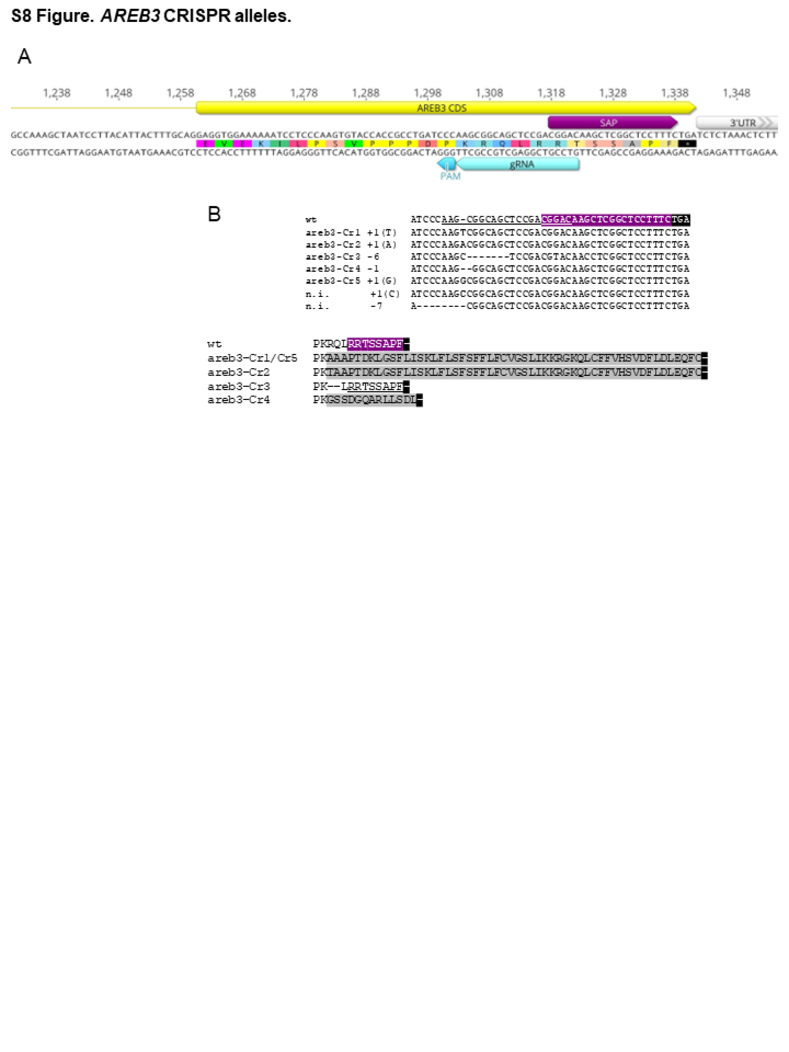

Supplement: S8 Fig — (A) Schematic representation of the third AREB3 exon with the position of the PAM sequence (blue), gRNA target (light blue) and the SAP motif (purple) (B) Genomic sequences of the CRISPR mutants isolated and their predicted protein sequences. The SAP motif sequence is highlighted in purple, the gRNA target sequence is underlined. 5 independent lines were isolated, and two more alleles were detected in T1 plants (see S9B Fig) but not isolated as homozygous lines (n.i.). The altered aminoacidic sequence of the mutants due to the frameshift mutation is highlighted in grey. Images produced using Geneious version 2022.0 created by Biomatters. (TIF) [file pgen.1010766.s011.tif]

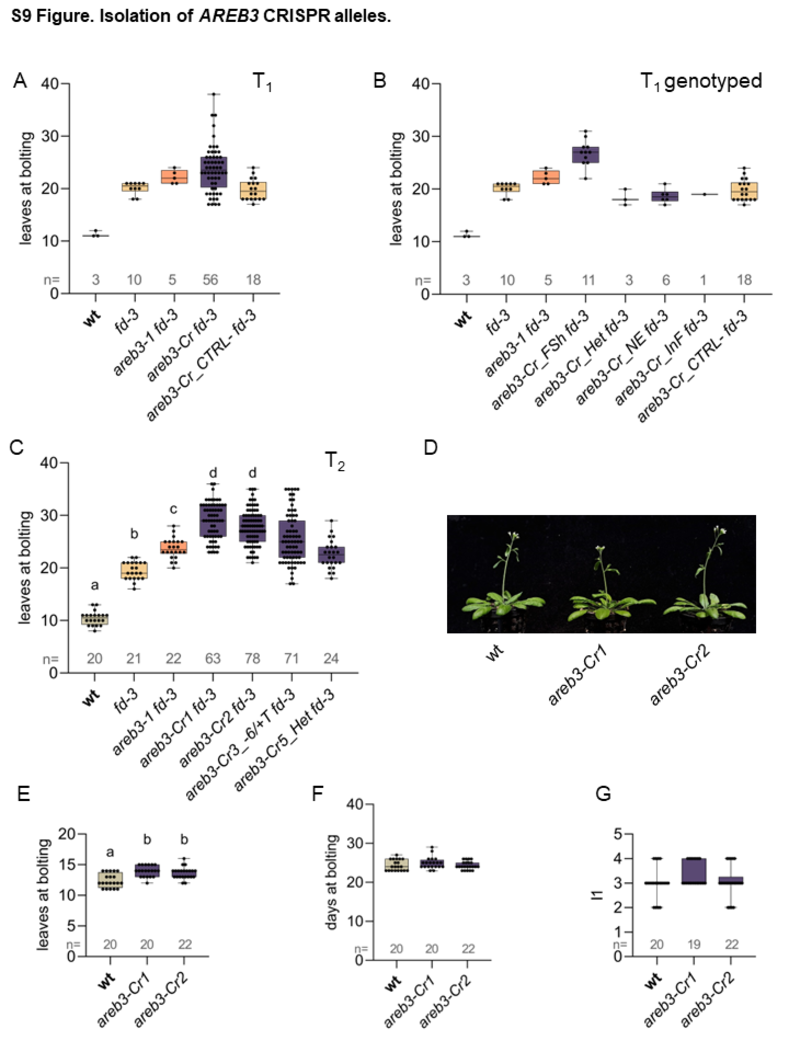

Supplement: S9 Fig — (A) T1: Flowering time expressed as number of rosette leaves of transgenic, RFP-selected T1 AREB3 CRISPR lines (areb3-Cr_T1 fd-3) compared with transformed non-transgenic RFP- control lines (areb3-Cr_CTRL- fd-3). (B) 21 T1 individuals genotyped by sequencing and categorized into the following groups: areb3-Cr_T1_FSh fd-3 (frameshift mutations); areb3-Cr_T1_Het fd-3 (heterozygous lines with one wt allele); areb3-Cr_T1_NE fd-3 (transgenic, not edited lines, both alleles are wt); areb3-Cr_T1_InF fd-3 (T1 line with an in-frame, -6 deletion in heterozygosity with a wt allele). (C) T2: Flowering time of RFP-, Cas9-free T2 independent AREB3 CRISPR mutant lines. Random individuals were genotyped by sequencing: areb3-Cr1_-1/+T fd-3 is a segregating population of biallelic frameshift mutants (-1/+T); areb3-Cr2 fd-3 is homozygous for a single nucleotide insertion (+A/+A), areb3-Cr3_-6/+T fd-3 is heterozygous for an in-frame, -6 deletion and a single nucleotide insertion (+T), and areb3-Cr5_Het fd-3 is heterozygous for a single nucleotide insertion (+G) and a wt allele (significance a vs b p = 8e-15; b vs c p = 6.67e-5; c vs d P<4.38e-7; p value on heterozygous populations not calculated). (D) The phenotype of areb3-Cr1 and areb3-Cr2 lines, obtained by backcrossing of the parental areb3-Cr1 fd-3 and areb3-Cr2 fd-3 lines with the wt at 4 weeks after sowing under LDs. (E) Flowering time expressed as number of rosette leaves of areb3-Cr1 and areb3-Cr2 lines (significance a vs b p<0.0005). (F) Days at bolting and (G) number of cauline leaves (I1 phase) of the indicated genotypes. No significant differences were observed. (TIF) [file pgen.1010766.s012.tif]

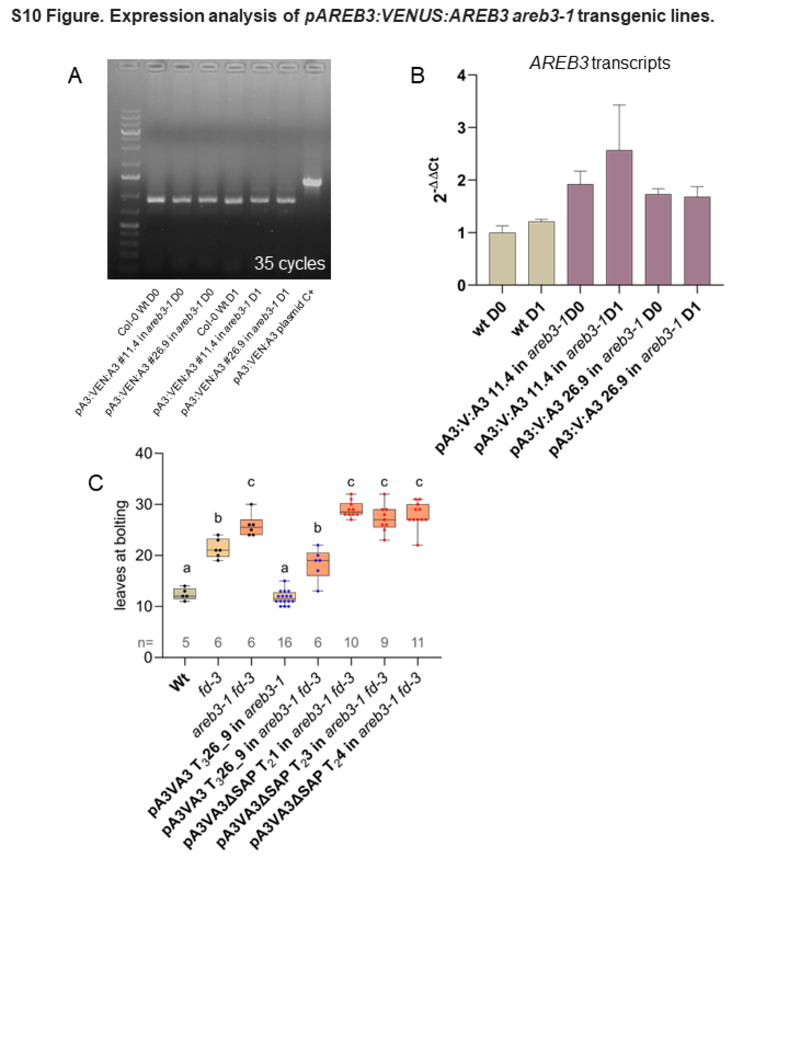

Supplement: S10 Fig — (A) RT-PCR showing the production of a complete, correctly spliced, transcript in the pAREB3:VENUS:AREB3 lines. (B) Real-time qPCR analysis of the same lines. Line 26.9 was chosen for subsequent experiments as the most similar to the expression levels of the wt. In both experiments, plants were grown for 2 weeks in SD (D0) before being moved to LD and resampled after one full day of LD (D1) at ZT8. (C) Flowering time analysis of areb3-1 mutant complemented with vector expressing either the complete AREB3 genomic sequence, or its truncated version lacking the last 24nt coding for the SAP motif. pA3VA3 T326_9 in areb3-1 is an isogenic pAREB3:VENUS:AREB3 line that was introgressed in the mutant areb3-1 fd-3. pA3VA3ΔSAP are independent pAREB3:VENUS:AREB3ΔSAP T2 lines in areb3-1 fd-3 (significance a vs b, c p<3.82e-4; b vs c p<9.09e-3). (TIF) [file pgen.1010766.s013.tif]

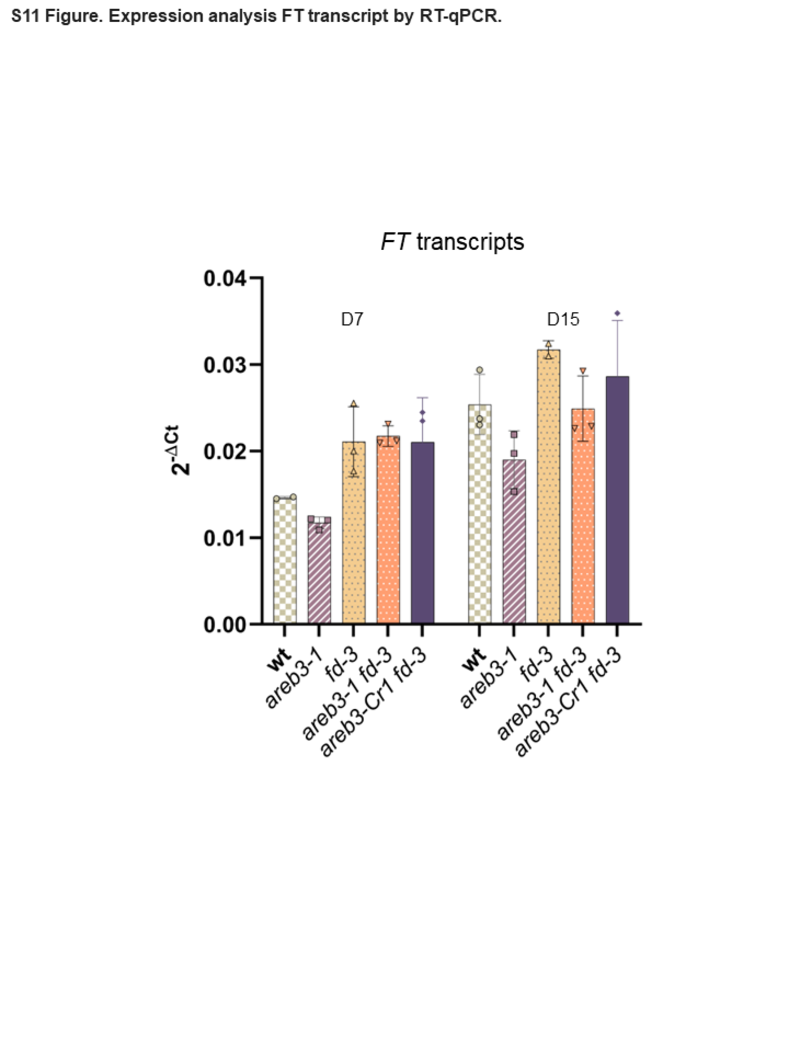

Supplement: S11 Fig — Plants were grown under LD conditions, and the above-ground plant material (rosette and hypocotyl) was collected at ZT16 at 7 days (D7) and 15 days (D15) after sowing, representing pre- and post-floral transition stages. Each point represents a pool of >3 individual plants. Mixed-effect analysis, Tukey’s multiple comparisons test evidenced no significant difference in all the tested data, excluding areb3-1 vs areb3-Cr1 fd-3 (p = 0.0313). One of two biological replicates is shown here. (TIF) [file pgen.1010766.s014.tif]

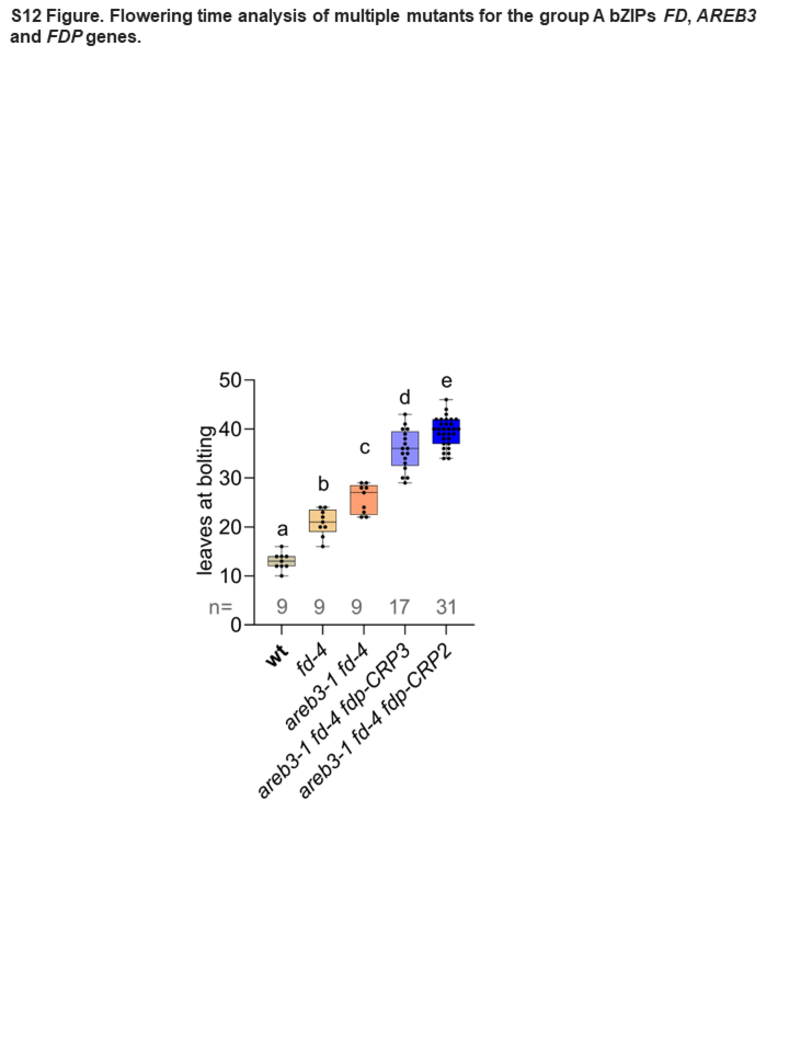

Supplement: S12 Fig — Plants were grown under LD conditions. Significance a vs b, c, d, e p<9.97e-06; b vs c p = 1.21e-02; c vs d p<1e-15; c vs e p = 4.52e-10; d vs e p = 2.14e-3. (TIF) [file pgen.1010766.s015.tif]
