# Supplementary material for: The bZIP transcription factor AREB3 mediates FT signalling and floral transition at the Arabidopsis shoot apical meristem
Source: PLoS Genet. 2023 May 15;19(5):e1010766. doi: 10.1371/journal.pgen.1010766 (PMC10212096; doi:10.1371/journal.pgen.1010766)
Supplement: Attachment — Submitted filename: Response letter.docx [file pgen.1010766.s016.docx]

Dear Dr Conti,

Thank you very much for submitting your Research Article entitled 'The bZIP transcription factor AREB3 mediates FT signalling and floral transition at the Arabidopsis shoot apical meristem' to PLOS Genetics.

The manuscript was fully evaluated at the editorial level and by two independent peer reviewers. The reviewers appreciated the attention to an important problem, but raised some substantial concerns about the current manuscript. The reviewer 1, in particular, asked for some more data of SAP domain function, to which we agree. Based on these reviews, we will unfortunately not be able to accept this version of the manuscript, but we would be willing to review a much-revised version. 

Should you decide to revise the manuscript for further consideration here, your revisions should address the specific points made by each reviewer. We will also require a detailed list of your responses to the review comments and a description of the changes you have made in the manuscript.

We are sorry that we cannot be more positive about your manuscript at this stage. Please do not hesitate to contact us if you have any concerns or questions.

Yours sincerely,

Li-Jia Qu
Section Editor
PLOS Genetics

*Dear Editor,*

*Thank you for your guidance with the submission of this new version of the manuscript, and I appreciate the time and effort that you and the reviewers have dedicated to providing us with valuable feedbacks.*

*In this new version we have been able to incorporate changes to reflect most of the suggestions provided by the reviewers. We include improvements to the text and important new data that strengthen our previous conclusions. We also updated the method section accordingly. All these changes are highlighted within the manuscript.*

*Finally, we provide a point-by-point response to the reviewers’ comments below (we highlight our response in italics for clarity).*

*We are confident that these new results have substantially increased the quality of our study and we look forward to hearing from you in due time regarding our submission and to respond to any further comments you may have.*

*Kind regards*

*Lucio Conti*

*PS Please note that in this new version we added an additional author - Paolo Korwin Krukowski. This was deemed appropriate for his contribution in generating new results included in this new version.*

Reviewer #1: In this manuscript Martignago et al., demonstrated the role of a transcription factor AREB3 in flowering time control by mediating FT signaling. The research started through a Y2H assay screening group A bZIPs that interact with florigen protein FT and TFL1. They found almost all of them interact with FT and TFL1 and have a SAP domain that has been shown functionally important in FD, a well know group A bZIPs TF in FT signaling. However, they continued with AREB3, the only one showing a canonical SAP except for the known FD and FDP. By genetic study and confocal imaging analysis they showed the functional redundancy of AREB3 with FD and FDP in FT signaling and TFL1’s antagonizing FT. The authors also generated a series CRISPR mutants of AREB3 to disrupt the SAP domain, which give strong genetic evidence supporting the functional importance of the SAP domain, however the biochemistry experiments didn’t show any influence from SAP deleted AREB3 on its physical interactions with FT and TFL1.

This is an interesting story which provides important clues regarding how hormones such as ABA is involved in flowering time control. The genetic analyses for the redundancy of between ABRE3 and FD& FDP and ABRE3’s function in FT signaling are fairly complete and great. However, whether the SAP domain is involved in this FT signaling is not clear. Therefore, I would expect more analyses on SAP domain function which will strengthen the conclusions made by the authors. The detailed comments are listed below.

*We appreciate the reviewer’s interest in our study and data.*

1. The narrative order about the SAP domain is a bit confusing. I suggest the assays about the SAP domain including both the genetic and biochemical parts (line 193-211 & line 149-172) to be integrated as an independent session and with some more analysis suggested below to be put in the last session.

*Thank you for this feedback. First, to ensure consistency with existing literature and avoid any confusion with a completely unrelated SAP domain involved in DNA damage signalling and repair, we opted to replace the term “SAP domain” with “SAP motif. As suggested, we organised the biochemical experiments/interaction assays related to the SAP motif under a new subheading (line 164). We also wish to emphasise that it was our comprehensive analysis of the SAP motif within the group A bZIP TFs that enabled us to identify AREB3 as the TF with the most similar SAP motif to FD (lines 149-172 in the first submission). This result prompted us to further characterise its role using genetics (lines 174-211 in the first submission). Within this context, we feel that the genetic analysis of the SAP motif should be placed after the description of the areb3 T-DNA lines, which demonstrates the biological relevance of AREB3. We see the Crispr-based effort as a validation of the idea that disrupting a putatively important motif of the bZIPs can cause equivalent phenotypic effects. Nevertheless, we have rephrased the text to make these ideas clearer to the readers, especially the ones describing why AREB3 was selected for further studies (lines 153-162 of the revised manuscript).*

2. To make it clear whether the SAP domain is involved in this FT signaling, at least one of the following assays need to be done:

1) The flowering phenotype of either pGAS1:FT areb3-Cr1 fd-3 or tfl1 areb3-Cr1 fd-3 (Cr1 can also be Cr2 or Cr4).

*Thanks for this valuable comment. While revising this manuscript, we obtained new flowering time data on areb3-Cr1/2 single mutants (S9 D-G Figure and line 217). These mutants show only very mild flowering time defects compared with the wild type. After weighing the potential benefits of revealing novel phenotypes against the time required to cross these Cr alleles with pGAS1:FT or tfl1, we have decided to focus our efforts on detecting the AREB3ΔSAP protein to support the potential co-existence of FT and AREB3^ΔSAP^ in Arabidopsis shoot meristem cells (please see below).*

2) The transient assays in tobacco leaves showing deletion of SAP domain doesn’t influence AREB3 FT/TFL1 interactions are not convincing, as the proteins are all overexpressed which may cause false positive.

*We agree with the reviewer about the potential problems associated with overexpression in heterologous systems. To mitigate this issue, we incorporated the* CONSTANS *protein as a negative control for the interaction with FT in our IPs assays. As expected, no protein interaction was detected (S3 Fig). Nevertheless, we fully acknowledge this caveat in the Discussion section (line 420).*

In vivo, the SAP domain may influence the protein stability or localization which might make the spatially co-existing of AREB3 and FT/TFL1 totally impossible. To this end, a VENUS:AREB3-ΔSAP is necessary to show both the abundance and spatial distribution of AREB3-ΔSAP is still the same with the WT AREB3. If not, the transient assay wouldn’t be useful to explain how SAP domain function.

*Thank you for suggesting this experiment. In the revised version of our manuscript, we include a detailed analysis of one T3 homozygous AREB3:VENUS:AREB3^ΔSAP^ line in the areb3-1 fd-3 background at different time points before and after floral transition. These new data (Fig 3B) show that, under the same confocal conditions used to detect VENUS:AREB3 in areb3-1 and areb3-1 fd-3 backgrounds, VENUS:AREB3^ΔSAP^ protein accumulates in nuclei and its pattern of expression in the SAM broadly reflects that of wild-type AREB3. This evidence led us to conclude that the AREB3^ΔSAP^ protein is available in the SAM cells/nuclei to interact with FT, although no downstream floral signal is activated (lines 253-255).*

3. It would be better to add some discussion about the canonical and noncanonical SAP domain. Any functional differentiations between?

*Thanks for this suggestion. The SAP motif is essentially a 14-3-3 recognition motif (Taoka et al. 2011; Nature). Based on a previous study (Johnson et al. 2010; Biochem J), plants have a conserved 14-3-3 binding site [LX(R/K)SX(pS/pT)XP] that is slightly different from the mode I canonical binding site identified for other species [R(S)XX(pS)X(P)]. We have included this information in lines 153-157 of the new manuscript version.*

*It is worth noting that at the time of the Johnson et al. study, only 14 plant 14-3-3 interacting proteins had been identified, and no group A bZIP proteins were included in the analysis. This may have influenced the definition of what is considered a “canonical” plant 14-3-3 binding motif. Despite that, FD presents a canonical plant 14-3-3 binding site at its C terminus (Fig. 1B), which was described as the florigen interaction site, and later as the 14-3-3 recognition motif (Abe et al. 2005, Science; Taoka et al. 2011, Nature). Indeed, the current model of the flowering activation complex (FAC) involves the group A bZIP TF FD, the florigen FT and the bridging 14-3-3 proteins, with the SAP motif having a key role for the formation and function of the complex. Within this context, the SAP motif is usually considered as a 14-3-3 recognition site present in the C terminus of group A bZIP TFs such as FD and FDP. Other group A bZIP TFs were shown to have similar 14-3-3 binding sites in their N-terminal region (called C1, C2 and C3 motifs in Furihata et al. 2006, PNAS). However, whether these motifs can be referred to as non-canonical SAP is uncertain and so is their role in flowering time regulation.*

*Considering the data available and the challenges associated with identifying canonical 14-3-3 binding sites in plants, we conclude that there is insufficient literature to distinguish the functional differences between canonical and non-canonical SAP motifs. Of course, we do agree that the role of canonical and non-canonical SAP motifs in flowering-time regulation is an important question, but outside of the scope of this study.*

*References*

*Abe M, Kobayashi Y, Yamamoto S, Daimon Y, Yamaguchi A, Ikeda Y, et al. FD, a bZIP protein mediating signals from the floral pathway integrator FT at the shoot apex. Science. 2005;309: 1052–1056. doi:10.1126/science.1115983*

*Johnson C, Crowther S, Stafford MJ, Campbell DG, Toth R, MacKintosh C. Bioinformatic and experimental survey of 14-3-3-binding sites. Biochem J. 2010 Mar 15;427(1):69-78. doi: 10.1042/BJ20091834. PMID: 20141511; PMCID: PMC2860806.*

*Furihata T, Maruyama K, Fujita Y, Umezawa T, Yoshida R, Shinozaki K, et al. Abscisic acid-dependent multisite phosphorylation regulates the activity of a transcription activator AREB1. Proc Natl Acad Sci U S A. 2006;103: 1988–1993. doi:10.1073/pnas.0505667103*

*Taoka KI, Ohki I, Tsuji H, Furuita K, Hayashi K, Yanase T, et al. 14-3-3 proteins act as intracellular receptors for rice Hd3a florigen. Nature. 2011;476: 332–335. doi:10.1038/nature10272*

4. Line 256-261, is there any evidence that ABRE3 protein level always consists with mRNA level during development? What makes it more unconvincing is that the ABRE3 mRNA is not detected in abre3-1 (no phenotype) but in abre3-1 fd-3 (additively late flowering). So the mRNA level may not be sufficient to explain the compensation for FD loss. Either a western blot for protein level of ABRE3 in fd-3 or quantification of fluorescence in figure3 is necessary.

*Thanks for raising this point. We first would like to clarify that although residual AREB3 expression is detectable in areb3-1 fd-3 double mutants, this level is much reduced compared with the fd-3 single mutant (S7 Figure). This suggests that the additive phenotype observed in areb3-1 fd-3 may derive from combined loss of FD and, in part, of AREB3 accumulation.*

*The major point raised by the reviewer concerns the question if increased RNA levels of AREB3 detected in fd translates into augmented AREB3 protein levels. To address this, we ran immunoblot experiments using isogenic lines AREB3:VENUS:AREB3 (areb3-1 or areb3-1 fd-3). We used similar conditions used in Fig 3D and extracted proteins from manually dissected apices of plants 3 days after the shift to LDs (ZT4-8). The results shown in Fig 3E indicate that there is an increase (1.6X) in AREB3 protein accumulation in fd-3 plants relative to the FD+ background. Therefore, we believe that this new result can strengthen the biological significance of the qPCR data presented in Fig 3D and convincingly support our argument that AREB3 accumulation is under feedback control by FD.*

5. Some data are missing or wrongly cited:

1) Line 213, no corresponding data were found in Figure S9, it seems should be S7, but also lack in S7.

2) Line 237, wrong citation, should be S9.

3) Line 293: citation is missing.

*Thanks for pointing us to these inconsistencies. We fixed all these issues and the provided the correct references to the corresponding Suppl Figures (which were in some cased renamed see point 3 below).*

6. Some figures and legends need to be improved to make is clearer:

1) Figure 1D, GFP:IP panel seems overexposured, could the it be performed with shorter exposure so the band is visible?

*As the immunoprecipitation targeted GFP-bound proteins, the IP part of the membrane probed with anti-GFP was indeed slightly overexposed. However, the goal of this part of the blot is to control that GFP-bound proteins were efficiently immunoprecipitated, which is the case. The membrane for the anti-MyC antibody is not overexposed and is the membrane that contains the co-IP results that formed the basis of our conclusions regarding the protein interactions. Similar results are shown in S3 Figure, which further validate the results presented in Fig 1D.*

Besides, is there any explanation why the sizes of the IPed bands of Myc-AREB3 and Myc-AREB3 S294A are different?

*Thanks for this comment. First, we have checked the sequences of our constructs to ensure that no mutation was present that could explain the observed migration. We thus presume that the loss in a phosphorylatable amino acid (T282A) might have affected protein mobility, which is often the case when proteins are subject to phosphorylation. While more biochemical work is needed to prove this hypothesis, the fact that both the wild type and T282A AREB3 proteins can be pulled down by FT or TFL1 suggests that phosphorylation status of the SAP motif does not seem to be critical to determine binding preferences with florigen-like proteins.*

2) Figure 1B, could the phosphorylated sites T282 and S294 indicated in the amino acid sequence?

*In this new version, we highlight the position of the phosphorylated amino acids within the SAP motif (Fig 1B).*

3) Figure S6 looks a bit mess and the complicated names of primers makes it so difficult to follow with the details. Strongly suggest to make it clearer.

*Thanks for this suggestion. We have divided the content of the original S6 Fig into two new supplementary figures (S6 and S8). The new S6 Fig describes the areb3 T-DNA genotypes used in this study, whereas S8 Fig focuses on the description of the Crispr alleles.*

4) Figures 3 & 4, what are 11 LD, 13 LD and 18LD FM et al. mean?

*Thanks for this suggestion. We have now included the meaning of LD in the legend.*

Reviewer #2: The manuscript by Martignago, da Silveira Falavigna, et al., entitled “The bZIP transcription factor AREB3 mediates FT signalling and floral transition at the Arabidopsis shoot apical meristem” reports the characterization of a FD paralog in Arabidopsis, AREB3, that can interact with FT and TFL1. Genetic analysis indicated that areb3 mutation enhanced the flowering phenotype of fd mutants. Floral transition is a topic of enormous significance, and this study reports a new player. They also showed an transcriptional compensation mechanism that has been recently found in plants (Rodriguez-Leal et al., Nat Genet 2019, 51:786–792). Overall, this is a solid study with conclusions supported by the data. I have only some minor comments.

*Thank you for your positive and encouraging feedback.*

1. P. 7, line 150, “We found that all group A bZIP genes presented at least one splicing form encoding highly conserved SAP domains within a previously described conserved carboxy-terminal region (Fig 1B [28]).” Line 153, “Besides FD and FDP, only AREB3 (also known as DPBF3, AtbZIP66, At3g56850) presented a canonical SAP motif.” It is confusing how AREB3 was selected. It seems that all bZIPs shown in Figure 1B have the SAP domain.

*The reviewer is correct in pointing out the conservation of the SAP motif within group A bZIPs and we made changes in the text to reflect this note (Lines 153-162). We also highlight that among all group A bZIP TFs, AREB3 presents the highest level of conservation of its C terminus in relation to FD/FDP (including the SAP motif – RTSSAPF in AREB3 vs. RSSTAPF in FD/FDP), which makes AREB3 a strong candidate for a more in-depth analysis.*

2. The authors hoped to use CRISPR to generate SAP domain deletion lines. However, frameshift may also interfere with gene expression. Are the truncated transcripts still expressed at levels comparable to the full-length version?

*We agree with the reviewer comment. Based on the RT-PCR results presented in S7 Figure we could detect similar levels of transcript derived from the AREB3 Cr1 and 3 alleles (fd background) with respect to the wild type AREB3 transcript (fd background). This indicates that the mutant transcript can accumulate in plant cells. We added a new RT-PCR result in S7 Figure - conducted with primer pairs that amplifies from the 3'-terminal end of the 1st exon and ending 124bp into the 3'UTR - which confirm the production of a complete, correctly spliced mRNA in the CRISPR mutants. We also suspect that these mutant allele-derived transcripts can be translated into correspondingly mutant proteins because of the clear detection of VENUS:AREB3^ΔSAP^ protein in nuclei (Fig 3B). Thus, the late flowering phenotypes of areb3-Cr alleles likely derives from impaired signalling downstream of FT/AREB3 complex formation.*

3. The roles of the SAP domain is confusing. On one hand, genetic analysis indicated that the SAP domain is required for AREB3 function. On the other hand, molecular assay indicated that SAP is not affecting AREB3 interaction with FT/TFL1. How to reconcile this discrepancy?

*Indeed, these results are puzzling and there is no consensus about the topic in the current literature, so we avoided to be over speculative in the discussion of our manuscript (Lines 410-424). Still, different reasons might explain these discrepancies: (i) the heterologous nature of several of the biochemical assays; (ii) if the function of the SAP motif is to provide protein stability, as suggested by Reviewer #1, a FT–AREB3^ΔSAP^ complex would be unstable and quickly degraded; (iii) conversely, a relatively stable FT–AREB3^ΔSAP^ could have an impaired ability to bind to DNA and to regulate gene expression. We believe that more biochemical data concerning the structure of the different protein complexes between group A bZIP TFs, FT/TFL1 and 14-3-3 proteins might shed light on this issue.*

________________________________________

Have all data underlying the figures and results presented in the manuscript been provided?

Large-scale datasets should be made available via a public repository as described in the PLOS Genetics data availability policy, and numerical data that underlies graphs or summary statistics should be provided in spreadsheet form as supporting information.

Reviewer #1: No: As discribed in the comments to the authors

Reviewer #2: Yes

________________________________________

PLOS authors have the option to publish the peer review history of their article (what does this mean?). If published, this will include your full peer review and any attached files.

If you choose “no”, your identity will remain anonymous but your review may still be made public.

Do you want your identity to be public for this peer review? For information about this choice, including consent withdrawal, please see our Privacy Policy.

Reviewer #1: No

Reviewer #2: Yes: Yuling Jiao
